# Alcohol: Its Therapeutical Uses, Internally and Externally

**Published:** 1881-09

**Authors:** Roberts Bartholow


### ALCOHOL: ITS THERAPEUTICAL USES, INTERNALLY AND EXTERNALLY.

By ROBERTS BARTHOLOW, M.D.

Of the general subject—alcohol—there has been assigned to me for this discussion its therapeutical uses, less the uses of malt liquors.

I have, therefore, to consider alcohol in the form of spirits—whisky, brandy, gin, etc.; of wines, still and sparkling, light and heavy, sweet and dry, grape juice and artificial, pure and fortified, etc. All of these agree in the presence of alcohol in varying proportions, but differ in respect to certain constituents, natural or factitious, and peculiar to each variety. It is obvious, therefore, that the question of the uses of alcohol is complicated by the numerous forms and combinations in which this substance enters the organism of man. In the short time allowed me I can do no more than present a syllabus of the subject—state merely a series of propositions.

Alcohol may be used as a stomachic tonic and to increase the activity of those functions included under the primary assimilation, as a cardiac and arterial stimulant, as an antipyretic, as an antiseptic, as an anodyne, and as a styptic and astringent. Besides these points we must consider whether it shall be given or withheld in mania a potu, or acute alcoholic delirium, and in delirium tremens. The subject may be best studied in this order.

I. As a stomachic tonic alcohol is effective only in the use of those not habituated to its use. As pepsin is not only precipitated by alcohol from its solution in the gastric juice, but is also rendered inactive as a ferment, to employ this agent as a means of promoting digestive activity is apparently paradoxical; but the explanation lies in the fact that it is a concentrated solution which acts thus on the pepsin. We obtain two points of practice from this statement—to give alcohol properly diluted; to stimulate digestion, administer the remedy immediately before meals: for the action of alcohol as a stomachic tonic consists in the power it has to cause a prompt congestion of the mucous membrane, and the increased activity of the gastric glands is a necessary consequence. That in time a catarrhal state of the mucous membrane is produced and a pathological secretion obtained, shows us the impropriety of the long-continued use of alcohol as a stomachic tonic, and explains why such a therapeutical effect is not the result of its administration in the subject of alcoholism.

In the atonic dyspepsia of the sedentary and the feeble digestion of the convalescent, alcohol is used in a high degree. The particular form of the remedy must vary with the peculiarities of each case. A wine of good body and decided boquet has been found very efficacious in the weak digestion of literary and nervous sedantary persons. In the case of the convalescent, a spirit is preferable to a wine, and whisky or brandy is prescribed according to individual tastes, partly, and the state of the intestinal canal—the former having a somewhat laxative, and the latter astringent, action. The quantity taken is determined by several considerations—by the amount of stimulation beneficial to the organs, by the effect as manifested in the circulatory and nervous systems, and by the degree of elemination. To the literary and nervous invalid class, the wine proposed is especially grateful, and they are responsive in a high degree. As the wine is to play the part of both stimulant to the digestive organs and a food of the nervous tissue, the best results are had from a generous wineglassful taken during the principal meal. The kind of wine selected is determined by several considerations. If the organs simply require stimulation, a good, dry sherry or Burgundy may be best adapted to the case; if an excess of acid be present, due to the fermentation of the saccharine and starchy foods, a sweet wine is objectionable, but then an acid Rhine wine of high alcoholic strength—as, for example, the Forster Riesling—may be serviceable in a remarkable degree. For the weak digestion of convalescence, it is well known that a considerable quantity of spirit is borne well and required. In both groups, but in an especial degree in the nervous and depressed invalid, the effect of alcoholic stimulation is peculiarly grateful and seductive, hence the danger of the alcoholic habit, and the need of circumspection in prescribing a remedy which may be so abused. When alcohol is used to promote the nutrition in general, it should be administered during and after meals, to obtain at the same time the effect due to stimulation of the gastric glands and the force derived from the oxidation of the remedy, whence it becomes in a certain limited sense a food. The amount requied in wasting diseases—as in chronic suppuration and in phthisis—will be more, and it will be better borne, than in other chronic maladies of a depressing kind; but in all chronic maladies the amount required is limited by two facts.: 1. A small quantity will stimulate the digestive organs without injuring the pepsin; 2. Only a small quantity can be oxidized, and therefore utilized as force. It follows that it is useless to attempt, by increasing quantity to overcome the inertia of the nutritive functions. Anstie, in the course of his well-known researches on the disposition of alcohol in the organism of man, found that beyond a certain small amount—two or three ounces of absolute alcohol—that which is taken is eliminated unchanged.

Applying these principles to phthisis—which may serve as an example of a large group of maladies—we may consider how and to what extent alcohol may be used in this disease. A notion prevails more or less widely among the laity, shared to some extent by the profesion, that alcohol is to a certain sense a prophylactic and curative agent in consumption. By some physicians, amylic alcohol is held to possess these powers. That these notions are not well founded is proved by the fact that so many subjects of chronic alcoholism die of phthisis. That beneficial results are obtained by the judicious use of alcohol is equally true. The questions for us to consider are how much alcohol is necessary, and what are the conditions of its use in consumption. The principles which I have already laid down are perfectly applicable. It is an axiom that when alcohol improves the appetite and increases the body-weight it is doing good. The quantity of alcohol sufficient to stimulate the gastric glands, and yet short of the strength necessary to precipitate the pepsin, is the proper quantity. As the alcohol eliminated by the various channels of excretion is in excess of the oxidizing powers of the organism, no benefit can result from such a quantity. It is obvious from these considerations that the best results are derived from half an ounce to an ounce of whisky or brandy, taken during or immediately after meals; for thus the stomachic tonic effects and the force-value of alcohol as a food is obtained. The now almost universal use of whisky with cod-liver oil is based on sound principles, unless, as is sometimes done, the mixture is given an hour or two before meals, which must have a disastrous effect upon the appetite and the digestion.

That there is any specific action of alcohol in consumption—that alcohol has an influence apart from its stomachic tonic effects and as a hydro-carbonaceous food—cannot for one moment be entertained. We may therefore dismiss the purely groundless assumption that amylic alcohol has some special virtues.

The use of alcohol in acute wasting diseases must come up hereafter.

In certain diseases of the digestive tube, alcohol in its various forms is highly useful. All the world knows that sometimes obstinate vomiting, not dependent on inflammation of the stomach, may be promptly checked by a tea to a tablespoonful of raw brandy. In the vomiting of uræmic intoxication, and especially of yellow fever, a dry champagne or our native sparkling wine may be very successful. A simple diarrhœa may be checked by brandy. Not to waste time in the recital of these trivial and well-known therapeutical properties, I pass on to the fact that in cholera infantum we have in brandy the most generally efficient curative agent. It is generally known, I believe, that infants bear a relatively larger quantity of alcoholic stimulants, but it is not known that large doses of brandy are singularly efficacious in the disease just mentioned. I have given half a teaspoonful to a teaspoonfull of cognac brandy every three hours to a child under one year, and above that to three years one to two teaspoonfuls. It should not be given with milk, but in water as warm as possible, and as remote from the time of food-taking as possible.

II. As a cardiac and arterial stimulant, alcohol is probably more frequently used than for any other purpose It may be well entitled "the remedy of paradoxes." It is employed in states of depression to give warmth to the surface and to increase the action of the heart and arteries, and in states of excitement of the vascular system and elevation of temperature it is used to lower both. This seeming paradox is readily explained; but the explanation must be reserved.

Alcohol is indicated when from any cause the action of the heart and of the arterial system is depressed suddenly and powerfully. The sudden failure of the heart from moral causes, from shock, from hemorrhage, from weakness of the cardiac muscle, are conditions in which alco hol is indicated, and in which it is used to advantage. There are two explanations of its power to elevate the heart and the vascular tension. The first is reflex. It is within the range of everybody's experience that as soon as a tablespoonful of whisky or brandy reaches the stomach the heart at once moves more rapidly—that is, the impression made on the end organs of the pneumogastric in the stomach reaching the pneumogastric nucleus, the inhibition exercised by the nerve relaxes, and the accelerated apparatus exercises its power with lessened hindrance. That this explanation applies with much probability is rendered more certain by the fact that the action of the heart is simply increased by alcohol; in other words, that the diastolic interval is shortened by its influence. The second explanation of the power of alcohol to increase the rate of the heart's movements is that by its oxidation force is generated, which is utilized by the cardiac ganglia. There are other substances, of course, which also yield force; but alcohol has the distinct advantage that its combustion occurs with more readiness than any other available substance. But we shall find hereafter that alcohol in large quantity depresses the circulation. Now comes the explanation of the apparent paradox. Whilst small doses stimulate the circulation, large doses have the opposite effect; therefore, our conclusion is, to overcome cardiac depression the stimulant must be administered in small quantity, but frequently. In many cases, a wine of good body, highly charged with œnanthic and other ethers, is preferable to a spirit.

At this point we should endeavor to arrive at some positive conclusion in regard to the administration of alcohol in preparation for the apprehended cardiac depresion of the anæsthetic state, or to relieve it. Professor H. B. Sands, M.D., of New York, has followed the practice for several years of inducing alcoholic intoxication as a preparation for the administration of ether or chloroform and to prevent shock. It is always inculcated to administer an ounce or two of whisky as a preliminary to the anæsthesia and to the operation. This may or may not be good practice.. It is good practice if the heart is sound and the full anæsthetic state is desired; it is bad practice if profound and protracted anæsthesia is not necessary, and if any cardiac weakness exist; for alcohol being the source when the anæsthetics are obtained, and a congener as regards their action on the nerve-centres and on the vascular apparatus, it must intensify their effects. The theoretical deduction is abundantly confirmed by clenical experience. In a recent discussion before this society, our learned colleague, Dr. Turnbull, strongly criticised the subcutaneous injection of alcohol in the case of lethal symptoms caused by ether, and he was sustained by the eminent therapeutist Professor H. C. Wood, and myself.

To inject brandy or whisky subcutaneously with a view to stimulate the respiratory and cardiac centres in chloroform or ether narcosis is therefore improper—contributes, indeed to the fatal result.

III. The antipyretic effects of alcohol involve more doubtful questions than those therapeutical effects heretofore considered, but the weight of authority is unquestionably in favor of the view that alcohol in large doses depresses the temperature. It is not my province to discuss the physiological data on which modern scientific opinion rests, but the practical considerations governing its use in disease. I start with the proposition—the elements of which is too often confounded—that the antipyretic effects of alcohol are distinct from its stimulant and and supporting properties. In the treatment of acute inflammations and fevers we may employ alcohol as an antipyretic, to depress the abnormal temperature, or as a support to the circulatory system and a stimulant to the digestive function. In the former case large, in the latter small, doses are necessary.

The modern conception of the fever process and the relation of high temperature to cardiac and cerebral paralysis have imparted a new and more powerful significance to the use of antipyretics. It may be compendiously stated, without going into the reasons for the statement, that alcohol does not compare with the cold bath, with quinia, even with digitalis, as an antypyretic. It is true Binz has apparently demonstrated its utility in the hyperpyrexia of pyæmia, but this fact does not invalidate the general proposition. There can be no question, however, regarding the utility of alcohol in the adynamic state in fevers and in inflammations. It is not, however, as a febrifuge, as a depressant of the temperature, as an antipyretic, that it is useful. It may be affirmed that in all cases of the feverish state, if digestion still goes on, if cardiac paralysis is not threatened, alcoholic stimulants are unnecessary. The two conditions demanding the administration of alcohol are failing digestion and failing heart. It is rare indeed that more than two ounces of wine or an ounce of whisky is required, even in the most pronounced adynamia. If this do not stimulate the stomach to better work, or itself furnish needed force to the failing heart, a larger quantity can do no more. How often do we see the stomach filled to overflowing with stimulants, and yet the the heart does not respond, because the smallest amount is not appropriated! When a thimbleful cannot be oxidized, why put into the stomach a pint? Graves formulated some admirable rules for our guidance in the use of alcoholic stimulants in fevers. Alcohol does good when the tongue changes from dry to moist, when the stomach can receive and digest more food, when the pulse declines in rapidity and gains in force, when the surface grows moist and cool from hot and dry, when the delirium ceases and an expression of intelligence replaces blank stupor.

In the event of sudden and alarming depression, digestion being suspended, it is possible to maintain the body-forces on alcohol alone. Very numerous examples confirm the conclusions of experiment, that the oxidation of alcohol furnishes the force necessary to maintain the vital operations for a number of days; but it should be definitely understood that in the administration of alcohol for this purpose no provision is made for the necessary processes of wear, and hence their must early come a termination to a life so maintained.

IV. Some recent experience justifies the belief that the antiseptic properties of alcohol may be made available in practice. That it is inimical to animal ferments, to the lower forms of life, and prevents or arrests putrefactive decomposition, are well known facts. That these properties may explain the effects of full doses of alcohol in diphtheria seems probable, for besides its influence over the cardiac depression, the micrococci colonies may be attacked by it as they migrate. In what way we may explain the results, it seems plain that alcohol, when pushed to a degree which seems somewhat extravagant, has appeared to exert a decided curative influence which cannot be explained by its stimulant and antipyretic action. From this point of view may be regarded the good effects of alcohol in the septic maladies—septicæmia, erysipelas, puerperal fever, and the exanthemata, notably smallpox. It seems to me beyond question that the undeniable good effects of alcohol in these septic diseases is not explained by referring the results to its other properties. If this be admitted, it follows as a necessary consequence that to be effective in these diseases alcohol must be administered in large doses. There must be maintained, in a certain sense, a saturation of the blood in order to act on the *materies morbi* efficiently. There must be, of course, a judicious measure of the force of the disease, and the doses of the remedy be made to conform. In my own experience I have found these principles a valuable guide to the treatment of this important group of diseases.

V. I need not detain you long on the subject of alcohol as an anodyne. It quite yields in importance to the valuable derivatives ether, chloroform, chloral, etc. It has probably yielded quite too absolutely to the anæsthetics so called, and might be used advantageously to benumb the sensibility to pain in the minor operations. It cannot be prescribed with safety for the relief of nerve-pain, since the alcohol habit is sure to be formed. As a temporary expedient it may be useful. A tablespoonful of raw brandy or whisky may arrest an attack of migraine impending. An anæmic headache or the wakefulness of anæmia may be quickly removed by alcoholic stimulation. It is a curious fact, true also of morphia, that when after the long continued use of spirit for neuralgia the remedy is discontinued the pain returns.

VI. What is the proper practice regarding the administration of alcohol in certain maladies caused by it? To render my position perfectly clear I must precede the answer to this question by some definitions. Magnan makes a distinction between acute alcoholic delirium and delirium tremens—the former a delirium due to the impression of alcohol on a brain not accustomed to it; the latter a delirium occurring in the subject of chronic alcoholism. In my "Treatise on the Practice of Medicine" (2d ed., p. 843) I have adopted the old but expressive terms *mani a potu* and *delirium tremens*—the former corresponding to Magnan's acute alcoholic delirium. I must insist on the distinction between these forms of alcoholic delirium; for the practice pursued is a necessary consequence. Now, as in mania a potu the delirium is a direct result of the alcoholic excess, the first step in the cure consists in the withdrawal of the alcohol. The practice should be different in most cases of delirium tremens. It is true, in a small propotion of cases the outbreak of delirium is due to sudden excess, but usually the opposite condition obtains. Owing to the failure of the stomach, neither food nor stimulant can be retained, and then the peculiar illusions and hallucinations appear. In such cases, also, the food, when the stomach is prepared to receive it, may fail to be digested, because the gastric glands, so long accustomed to the stimulation of alcohol, will not act in its absence. It is supprising how in these cases, after some preparatory treatment, when food is taken with more or less stimulant, sleep and sanity follow expeditiously, without the intervention of doubtful hypnotics.

Alcohol performs another role in those cases of delirium tremens complicated with croupous pneumonia, or cases of croupous pneumonia complicated with delirium. With the progress of the pulmonary obstruction, ischæmia of the arterial system increases, and hence the laboring heart needs the support which alcohol may supply. Thus having correct principles to guide our treatment of delirium tremens, the vexed question of the use of alcohol in this disease is readily solved.

VII. The power of alcohol to coagulate albumen, to suspend the activity of the unorganized ferments, and to destroy minute organisms, lies at the foundation of its external uses. It is a most efficient hæmostatic to restrain bleeding from wounded surfaces, as I have repeatedly verified. As an antiseptic dressing to wounds, to prevent the entrance of the germs of putrefaction, to check suppuration, and to promote healing, it has in my experience scarcely been inferior to the much-vaunted carbolic acid. It is an efficient means for procuring local refrigeration of an inflamed joint or swelling Injected under the skin in the neighborhood of painful nerves, it has no inconsiderable anodyne power. This property may be utilized for the relief of myalgia and lumbago. A few drops thrown into the affected tussues will usually afford permanent relief. It is more efficient than water, used by the method now known as aquapuncture. Enlarged tonsils, hypertophied thyroid, and grandular swellings may often be slowly reduced and made to disappear by the parenchymatous injection of alcohol. This method is applicable to the treatment of uterine fibroids.

In cases of sudden depression of the powers of life, whisky may be thrown under the skin, to obtain more speedily than by the stomach its effect as a cardiac stimulant. I have already mentioned the contra-indication of this practice in the treatment of the narcosis or the cardiac depression caused by the anæsthetic agents ether and chloroform. It is, however, perfectly applicable to other conditions characterized by sudden and powerful depression of the heart. The amount injected varies from ten minims to a drachm; a syringeful—about half a drachm—is usually inserted at once, and is repeated according to necessity. Some local swelling arises, which may subside in a day or two, or an indurated lump remain for a time, or rarely suppuration may occur.—*Philadelphia Medical Times.*

